# Time- and concentration-dependent changes in gene expression induced by benzo(a)pyrene in two human cell lines, MCF-7 and HepG2

**DOI:** 10.1186/1471-2164-7-260

**Published:** 2006-10-16

**Authors:** Sarah L Hockley, Volker M Arlt, Daniel Brewer, Ian Giddings, David H Phillips

**Affiliations:** 1 Section of Molecular Carcinogenesis, Institute of Cancer Research, Brookes Lawley Building, Cotswold Road, Sutton, Surrey SM2 5NG, UK; 2 Cancer Research UK DNA Microarray Facility, Institute of Cancer Research, Cotswold Road, Sutton, Surrey, SM2 5NG, UK

## Abstract

**Background:**

The multi-step process of carcinogenesis can be more fully understood by characterizing gene expression changes induced in cells by carcinogens. In this study, expression microarrays were used to monitor the activity of 18,224 cDNA clones in MCF-7 and HepG2 cells exposed to the carcinogen benzo(a)pyrene (BaP) or its non-carcinogenic isomer benzo(e)pyrene (BeP). Time and concentration gene expression effects of BaP exposure have been assessed and linked to other measures of cellular stress to aid in the identification of novel genes/pathways involved in the cellular response to genotoxic carcinogens.

**Results:**

BaP (0.25–5.0 μM; 6–48 h exposure) modulated 202 clones in MCF-7 cells and 127 in HepG2 cells, including 27 that were altered in both. In contrast, BeP did not induce consistent gene expression changes at the same concentrations. Significant time- and concentration-dependent responses to BaP were seen in both cell lines. Expression changes observed in both cell lines included genes involved in xenobiotic metabolism (e.g., *CYP1B1*, *NQO1*, *MGST1*, *AKR1C1*, *AKR1C3*,*CPM*), cell cycle regulation (e.g., *CDKN1A*), apoptosis/anti-apoptosis (e.g., *BAX*, *IER3*), chromatin assembly (e.g., histone genes), and oxidative stress response (e.g., *TXNRD1*). RTqPCR was used to validate microarray data. Phenotypic anchoring of the expression data to DNA adduct levels detected by ^32^P-postlabelling, cell cycle data and p53 protein expression identified a number of genes that are linked to these biological outcomes, thereby strengthening the identification of target genes. The overall response to BaP consisted of up-regulation of tumour suppressor genes and down-regulation of oncogenes promoting cell cycle arrest and apoptosis. Anti-apoptotic signalling that may increase cell survival and promote tumourigenesis was also evident.

**Conclusion:**

This study has further characterised the gene expression response of human cells after genotoxic insult, induced after exposure to concentrations of BaP that result in minimal cytotoxicity. We have demonstrated that investigating the time and concentration effect of a carcinogen on gene expression related to other biological end-points gives greater insight into cellular responses to such compounds and strengthens the identification of target genes.

## Background

The DNA damage that is caused by chemical carcinogens is important in the initiation of carcinogenesis. For promotion and progression of an initiated cell to occur, however, other events within the cell need to take place and such events are likely to involve gene expression changes induced by the carcinogen. A broader understanding of the impact of carcinogen treatment in specific cells can be mechanistically informative and may enlarge the number of candidate genes contributing to variations in individual susceptibility to carcinogens. Microarray technology offers an attractive method by which to look globally at the extent to which gene expression is affected by carcinogen exposure and may give key insights into its carcinogenic effects [1-3].

The prime aim of this study was to look at early gene expression changes induced by the environmental carcinogenic polycyclic aromatic hydrocarbon (PAH), benzo(a)pyrene (BaP) [4,5], at non-cytotoxic doses, in order to identify novel genes/pathways involved in the cellular response to genotoxic carcinogens. We also wanted to investigate if the gene expression data could be correlated with other cellular effects of carcinogen exposure. To date, reports concerning expression profiling in mammalian tissues and cells following exposure to BaP or its metabolites [3,6-11] have been limited by several factors, including the use of only one concentration or exposure time, investigation of only one cell type, and in many cases only small, targeted array-sets have been used, which can restrict the number of novel candidate genes that can be identified in such carcinogen-exposure experiments. The cellular response to genotoxic stress may depend on the cell type being insulted, compound concentration and duration of exposure and it is important to understand the common and specific pathways of such responses. In this study we have analysed the transcriptomes of two distinct human cell lines, MCF-7 derived from a breast carcinoma and HepG2 originating from a hepatocellular carcinoma, after exposure to multiple concentrations of BaP and for different lengths of time to identify the relationship between these variables and gene expression modulation. Both cell lines are known to be metabolically competent in bioactivating carcinogens such as BaP [3,6] and contain wild-type p53 alleles [12,13]. In order to gain as much mechanistic insight as possible and to get a global picture of gene expression we have used large cDNA microarrays allowing us to analyse a total of 18,224 known human genes and expressed sequence tags (ESTs). Phenotypically anchoring carcinogen-induced gene expression profiles to other measures of toxic/genotoxic insult can aid in the identification of target genes [11]. Gene expression data have therefore been related to a number of other phenotypic measures, including DNA adduct formation, cell cycle effects and p53 protein activation. By linking the gene expression changes to these biological outcomes we aim to strengthen the process of identifying target genes involved in the cellular response to BaP exposure. Cells were also exposed to the non-carcinogenic isomer of BaP, benzo(e)pyrene (BeP), to try to distinguish between the genotoxic and toxic gene expression responses to BaP.

Profiles of transcription signatures generated for HepG2 and MCF-7 cells were used to compare overall patterns of gene expression and to identify differentially expressed genes. Here we report on a number of BaP-induced gene expression changes identified as unique to each cell line, but also on a sub-set common to both cell lines. Our results suggest a complex gene expression response to BaP exposure in human cells; nevertheless, some clear time- and concentration-dependent gene expression responses were observed, highlighting the importance of using multiple concentrations and time-points in carcinogen-exposure gene expression studies. We have also shown that relating the expression data to other measures of cellular stress can give greater understanding of molecular mechanisms involved in the cellular response to carcinogen exposure.

## Results

### DNA adduct analysis

**Figure 1:**
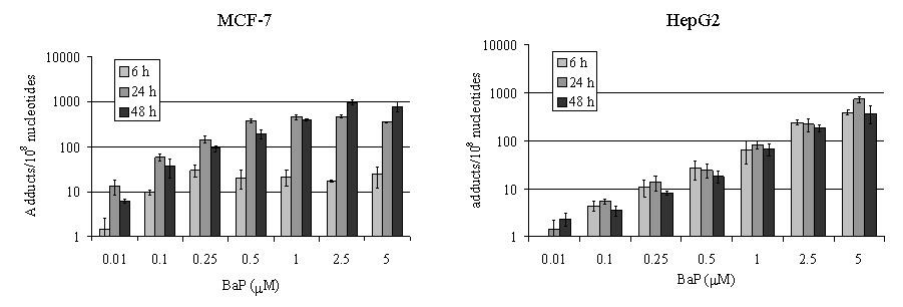
DNA adduct formation measured by ^32^P-postlabelling in MCF-7 cells and HepG2 cells after BaP exposure. The values are the log^10 ^mean ± S.D. of duplicate determinations of 2 independent experiments.

DNA adducts were measured in cells exposed to BaP for up to 48 h (Figure 1) in order to establish biologically significant concentrations to be used for the microarray experiments and to enable gene expression changes to be related to DNA damage levels. As shown in Figure 1, in MCF-7 cells BaP exposure resulted in a time- and concentration-dependent response in DNA adduct formation via the reactive metabolite anti-benzo(a)pyrene-*trans*-7,8-dihydrodiol-9,10-epoxide (BPDE) bonded to the *N*^2 ^position of guanine. A low level of DNA adduct formation was observed in MCF-7 cells after 6 h exposure relative to 24 and 48 h exposure. After 24 and 48 h exposure, DNA adduct levels increased with BaP concentration up to 1 μM at 24 h and to 2.5 μM at 48 h, after which a plateau was reached in this cell line. A concentration-dependent increase in adduct formation was also detected in HepG2 cells exposed to BaP (Figure 1), however, similar adduct levels were detected at each time-point. Adduct levels were generally lower in HepG2 cells than in MCF-7 cells, although the numbers of adducts in both cell lines did fall within the same order of magnitude. DNA adducts were not detected in MCF-7 or HepG2 control cells. Exposure of cells to 2.5 and 5.0 μM of BeP did not result in loss of cell viability or DNA adduct formation (data not shown).

**Figure 2:**
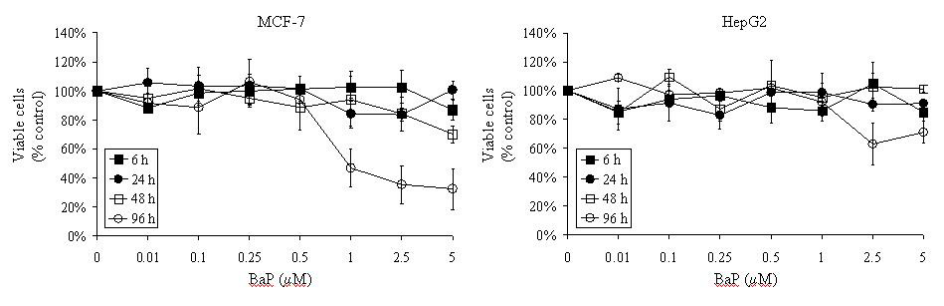
Effects of BaP on MCF-7 and HepG2 cell viability relative to controls. The values are the mean ± S.D. of two independent experiments.

BaP concentrations (0.25, 1.00, 2.50, 5.00 μM) were then chosen for the microarray experiments that resulted in low to high DNA adduct formation. A principle aim of this study was to identify gene expression changes related to carcinogenesis rather than toxicity. Therefore exposure times of up to 48 h were used as limited cytotoxicity was observed up to this time-point (Figure 2).

### Gene expression profiling of treated cells

**Figure 3:**
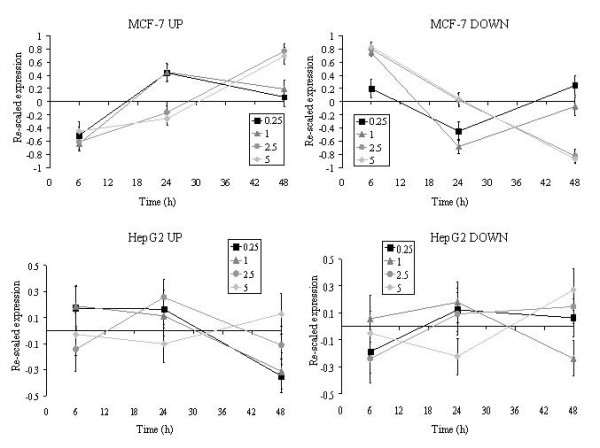
Average gene expression profiles for time- and concentration-dependent MCF-7 up-regulated genes and down-regulated genes and HepG2 up-regulated genes and down-regulated genesidentified from the 15 K microarray experiments after BaP exposure (0.25–5 μM). Each gene profile has been re-scaled so that its mean is equal to 0 with a variance of 1. These profiles represent the average shape of the gene expression profiles and error bars represent the S.E. of the mean.

**Figure 4:**
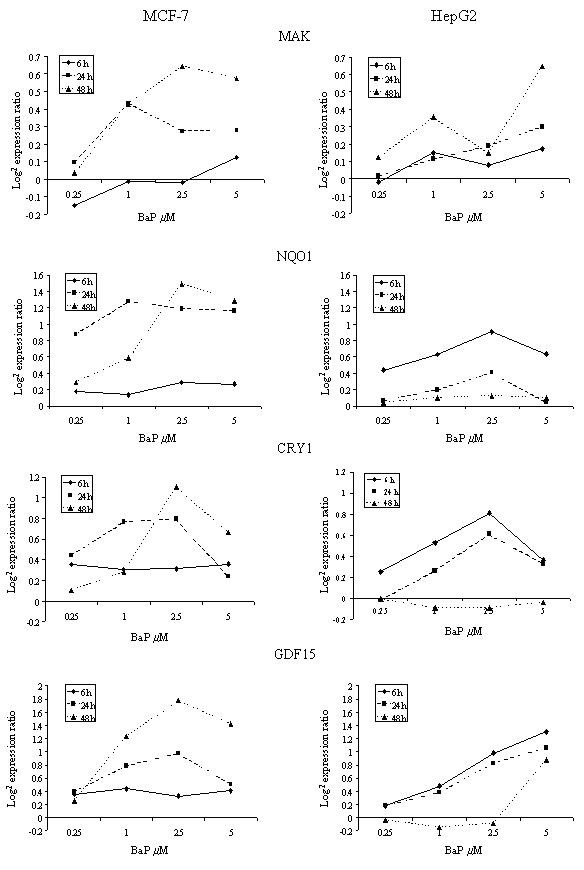
Example expression profiles of four genes affected by time and concentration in both MCF-7 (left column) and HepG2 cells (right column).

Filtering of Lowess normalised gene expression data in GeneSpring software identified 202 and 127 cDNA clones (See Additional file 1) that were modulated by at least 1.4-fold and that had a significant t-test *p*-value (< 0.05) in at least one sample of the MCF-7 and HepG2 cells, respectively. A number of altered genes were represented on both the 15 K and 6 K microarray systems and their consistent induction or repression on both arrays gives confidence in the reproducibility of the two systems. 2-Way ANOVA was performed on these gene lists in order to identify gene alterations dependent on BaP concentration or exposure time or both (Table 1 and Additional files 2 and 3). In both cell lines genes were altered in a time- and concentration-dependent manner and such expression changes are likely to be a true effect of BaP exposure. Further to this, the overall effect of time and concentration on BaP gene expression modulation in the two cell lines was analysed (Figure 3 and 4). Exposure of the MCF-7 cells to the lower concentrations of 0.25 and 1 μM BaP appears to result in initial induction or repression of gene expression after 24 h exposure followed by recovery to normal expression levels after 48 h (Figure 3). There is little effect on gene expression in the MCF-7 cells after 6 h exposure to the higher concentrations of 2.5 and 5 μM BaP, followed by induction after 24 and 48 h with greatest levels being reached at 48 h. For a number of genes affected by BaP in this cell line there is an increase in expression with concentration after 24 and 48 h exposure with a plateau being reached at 1 μM after 24 h and 2.5 μM after 48 h, examples of which can be seen in Figure 4. This response mirrors the DNA adduct formation response observed in the MCF-7 cells (Figure 1). In Figure 3 it can be seen that clear induction of expression in the HepG2 cells is only evident for 5 μM exposure at 48 h. For the lower concentrations the general pattern looks to be initial induction or repression at the earlier time-points of 6 and 24 h followed by recovery to normal expression at 48 h (Figure 3). Expression levels of a number of genes in this cell line increase with concentration although a plateau is reached at 2.5 μM for some of the genes, examples of which can be seen in Figure 4. The HepG2 gene expression response does not correlate so well with the DNA adduct response although the earlier expression response in the HepG2 cells is consistent with the higher adduct formation that occurs after 6 h BaP exposure in this cell line relative to MCF-7 cells. In both cell lines the degree of gene modulation was small with fold-changes rarely exceeding 3-fold. The number of changes identified was also relatively low with less than 1% of the arrays being modulated and this is consistent with other microarray studies that have looked at the effects of BaP [6,14].

**Figure 5:**
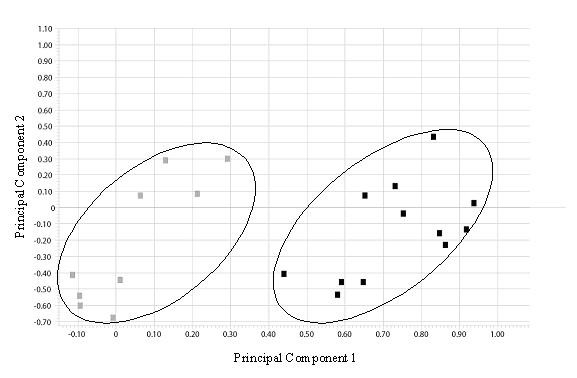
Principal component analysis of MCF-7 cells exposed to BaP and BeP using the 165 BaP-modulated genes from the 15 K microarray analysis. Black represents BaP samples and grey, BeP samples.

MCF-7 and HepG2 cells were also exposed to the non-carcinogenic isomer of BaP, BeP, which does not form DNA adducts, in order to distinguish genotoxic from toxic responses induced by BaP. MCF-7 cells were exposed to up to 2.5 μM BeP for 6, 24 and 48 h and HepG2 cells to 5 μM for 6 and 24 h. Gene expression changes induced by BeP were analysed using the 15 K cDNA microarray. In both cell lines few genes were significantly altered over 1.4-fold by BeP (Additional file 4) and comparison of the BaP and BeP expression data did not identify any common significant or consistent gene changes. Figure 5 illustrates that the BaP and BeP gene expression responses are clearly different.

In this report we have concentrated on those genes whose expression was altered according to time or concentration and also on those common between the two cell lines as these are more likely to be linked to carcinogenesis. EASE analysis [15] was performed on genes identified by 2-Way ANOVA as affected by time and concentration to find biological processes significantly over-represented in these gene lists for the two cell lines in order to identify any biological themes that occur in response to BaP. Biological processes significantly affected (Fisher exact test p value < 0.05) by BaP are listed in Tables 2, 3, 4 and Additional file 5. In both cell lines BaP down-regulated a number of processes related to nucleosome assembly and chromatin structure organisation. In the HepG2 cells these processes were both up-regulated and down-regulated. Over-represented themes among the up-regulated genes in both cell lines included oncogenesis and cell cycle progression from G2 to M phase.

**Figure 6:**
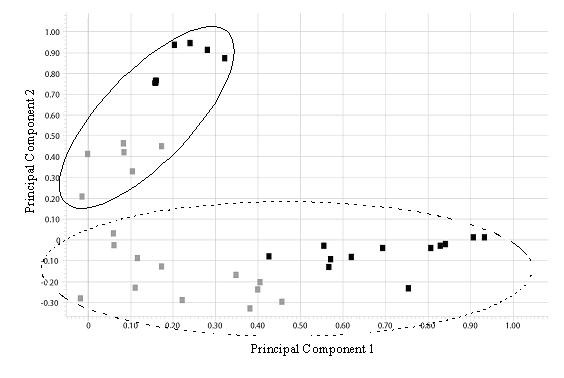
Principal component analysis of MCF-7 and HepG2 cells exposed to BaP using the total BaP-modulated genes in both cell lines from the 15 K and 6 K microarray analysis (302 genes). Black represents MCF-7 samples and grey, HepG2 samples. Samples grouped by the solid line are 6 K samples and those by the dashed line are 15 K samples.

Comparison of the MCF-7 and HepG2 BaP gene lists identified 27 genes as being modulated in both cell lines (Table 5). All genes were modulated in the same direction in both cell lines with the exception of *CRADD*. Also, in the HepG2 cells, five histone genes that function in nucleosome assembly, together with *TAF6L *and *UNC84A*, were repressed at 6 h, then induced at 24 h. In comparison, these genes were repressed, but not induced, in the MCF-7 cells and only after 48 h BaP exposure. The relationship between the MCF-7 and HepG2 cells' expression signatures was visualised by Principal Components Analysis (PCA) (Figure 6). This illustrates that there is greater similarity between the expression profiles of the two cell lines than there is between technical differences in the experiment (i.e. the two different microarray formats). DNA adduct levels were correlated with the expression changes of the 27 common genes (Table 5). In the MCF-7 cells 21/27 genes significantly correlated with DNA adduct levels and only 8/27 did so in the HepG2 cells. A number of genes correlated negatively with DNA adduct levels in the MCF-7 cells, including the five histone genes involved in nucleosome assembly.

### RTqPCR

RTqPCR, a more sensitive and specific measure of gene expression, was carried out to validate a number of interesting expression changes and to determine the reliability of the microarrays. Eight well-established and relevant genes were selected to be measured by RTqPCR. Seven genes that were up-regulated on the microarrays were analysed in this way (*CYP1B1, NQO1, AKR1C3, BAX, PCNA, CDKN1A *and *IER3*) along with one down-regulated gene (*HIST1H3D*). In the majority of cases the RTqPCR data confirmed, and correlated significantly with, the microarray data (Table 6) although expression ratios were generally underestimated by the microarrays relative to the RTqPCR (Additional file 6). For *CYP1B1*, however, correlation between the two methods was very low for both cell lines. In the HepG2 cells no change in this transcript was clearly evident from the microarrays, whereas RTqPCR identified strong induction. RTqPCR was also used to measure the expression of two genes, *CYP1A1 *and *AHR*, encoding proteins widely known to be involved in the cellular response to BaP, but not present on the microarrays. RTqPCR results displayed strong induction of *CYP1A1 *in both cell lines while the expression of *AHR *was not altered (Additional file 6). Pearson correlation analysis of DNA adduct levels with the microarray and RTqPCR data shows that RTqPCR measurement of gene expression generally correlates better with the adduct levels, confirming its utility as a more sensitive measure of gene expression. RTqPCR was also used to measure the expression of *CDKN1A*, *CYP1A1*, *CYP1B1*, *HIST1H3D *and *IER3 *in BeP-treated MCF-7 and HepG2 cells. This method confirmed the microarray results showing that this PAH did not alter the expression of these genes (data not shown).

### p53 protein expression

**Figure 7:**
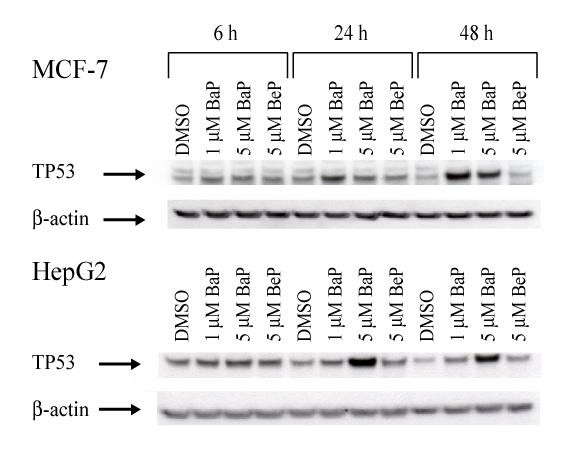
Changes in p53 protein level in MCF-7 cells and HepG2 cells in response to BaP and BeP detected by Western blot. β-actin antibody was used to detect β-actin protein expression, which was used as loading control.

**Figure 8:**
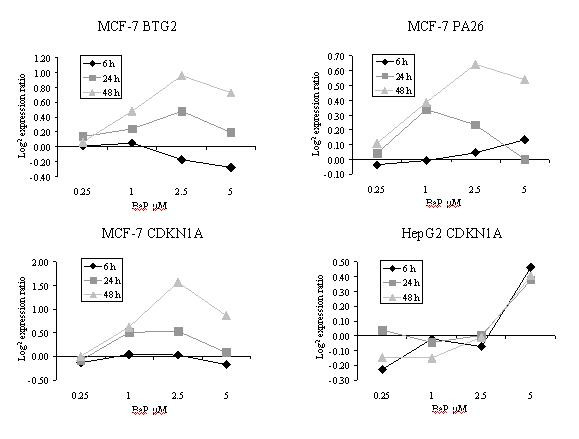
Gene expression profiles of a selection of p53-regulated genes induced in MCF-7 cells and HepG2 cells.

A number of p53-regulated genes were modulated in response to BaP exposure (*CDKN1A *and *BAX *in both MCF-7 and HepG2 cells and also *BTG2*, *PA26*, *CCNG1*, and *DDB2 *in MCF-7 cells). As expected, induction of p53 gene expression was not observed on the microarrays and this was confirmed by RTq-PCR (Additional file 6). p53 protein levels were assessed by Western blot in order to confirm accumulation of this tumour suppressor in response to the BaP concentrations used in this study (Figure 7). Protein levels were analysed in MCF-7 and HepG2 cells incubated with 1 and 5 μM BaP or 5 μM BeP for 6, 24 and 48 h and compared to control cells exposed to DMSO alone. An increase in p53 protein was observed in MCF-7 cells after exposure to 1 and 5 μM BaP for 24 and 48 h but not 6 h, with higher levels being observed after 1 μM exposure. Induction of p53 protein was observed in the HepG2 cells only after incubation with 5 μM at 24 and 48 h. These profiles of p53 protein activation agree with the expression patterns of the p53-regulated genes in the two cell lines, examples of which can be seen in Figure 8. In the MCF-7 cells induction of these genes was observed after 24 and 48 h but not 6 h, with expression increasing with time and lower levels of induction observed after 5 μM exposure relative to 1 μM. Induction of *CDKN1A *in the HepG2 cells was only observed after 5 μM treatment, in agreement with p53 protein accumulation in this cell line, although up-regulation of this gene occurred at all time-points, whereas protein accumulation was only seen at 24 and 48 h. No increase in p53 protein was observed after exposure to 5 μM BeP in either cell line.

### Cell-cycle analysis of MCF-7 and HepG2 cells exposed to BaP

The identification of altered expression of transcripts that may effect cell cycle regulation and genes whose expression is tightly coupled with DNA synthesis on the microarrays prompted the investigation of the effects of BaP on the cell cycle parameters of MCF-7 and HepG2 cells. Data representing the mean of two independent experiments are collated in Table 7. Over time, both DMSO- and BaP-treated cells appear to accumulate in the G1 phase with a corresponding decrease in the number of cells in S phase, which is indicative of cells reaching confluence. This is in agreement with the detection of increasing numbers of cells over time to form a confluent monolayer in the medium (data not shown). In both cell systems, there was marginal accumulation of cells in the S phase of the cell cycle after 24 and 48 h exposure to 2.5 μM BaP as compared to DMSO-treated cells. By 72 h the cells had resumed moving through S phase with a marginal accumulation in G1 occurring in the MCF-7 cells and an accumulation in G2 occurring in HepG2 cells. Arrest of the cells in the S phase of the cell cycle implies that DNA synthesis is being inhibited in both cell lines in response to BaP exposure.

## Discussion

Chemical carcinogenesis is a multi-step process still not fully understood and the identification of environmental factors that influence this process and insight into their mechanistic action will help our understanding of cancer and its causes, and ultimately how it can be prevented. Studies have shown that microarray technology is a powerful tool for identifying gene expression patterns that are reflective of a cell's response to chemical exposure [3,7,16] and can give insight into mechanism of action [1,17]. The aim of this study was to evaluate the use of this technology to elucidate the cellular response to a carcinogenic PAH, BaP, in mammalian cells, in order to identify genes/pathways involved in the carcinogenic process and to assess how such expression profiles relate to other phenotypic measures of BaP exposure.

Several studies have now looked at the effects of BaP or its metabolites on mammalian gene expression [6-10]; they have been limited, however, by the use of only one time-point or exposure concentration, have investigated only one cell type or have used DNA microarrays with a limited number of genes. To gain a more complete picture of the BaP gene expression response we have used multiple exposure times and concentrations, assessed two different cell systems, and used large genome-wide microarrays containing 18,224 cDNA clones. In addition, the gene expression profiles have been anchored to other phenotypic measures of BaP exposure. DNA adduct measurements, cell cycle analysis and p53 protein expression have been investigated and gene expression changes have been linked to these biological outcomes. The combining of data from these different measures permits a more complete understanding of the biological effects of BaP on human cells. MCF-7 and HepG2 cells have been used in this study based on their widespread use in toxicity studies and/or gene expression studies [3,6,18]. Although these are not normal cells they have the advantage of being easy to maintain, are well-characterised, and have normal p53 function. In addition, regulation of xenobiotic metabolising genes has been shown to be similar in HepG2 and primary human hepatocytes [19], suggesting that the former are a suitable tool to study gene regulation in liver cells.

A number of time- and concentration-dependent gene expression changes were identified in both cell lines in response to BaP exposure (Table 1), although the effect of these parameters on the gene expression profiles differed between the MCF-7 and HepG2 cells (Figure 3 and 4). Genes altered in a time- and/or concentration-dependent manner are likely to be true effects of BaP exposure. A select list of genes identified by Mahadevan et al. [6] as altered by BaP in MCF-7 cells, using the Affymetrix oligonucleotide U133A human genome array representing over 22,000 genes, showed overlap with the MCF-7 gene expression changes identified in this study, including *CYP1B1*, *AKR1C1*, *CDKN1A*, *BAX*, *DDB2 *and *NQO1*. This increases confidence to our conclusions and that the effect is strong enough to be picked up with multiple techniques.

Although the majority of expression changes identified were cell type specific, 27 genes were identified as altered in both cell lines (Table 5), indicating that BaP induces a general gene expression response in different cell types, although the timing of modulation is different. These common genes had functions that include xenobiotic metabolism (MGST1, NQO1, AKR1C1, AKR1C3 and CPM), cell cycle regulation (CDKN1A), nucleosome assembly (HIST1H2BG, HIST1H4B, HIST1H2BJ, HIST1H3D and HIST1H4C), anti-apoptosis (IER3) and oxidative stress response (TXNRD1). Since phenotypic measures such as DNA adduct levels and cell viability were similar in both cell lines it is likely that these common gene expression changes are related to carcinogenesis and may represent potential biomarkers of genotoxic insult.

We hypothesised that comparing the expression pattern of BaP to a non-carcinogen would aid in the identification of genes involved in carcinogenesis. Exposure of cells to equimolar concentrations of the non-carcinogenic PAH, BeP, had only a modest effect on gene expression in both cell lines and no consistent or significant gene alterations in common with BaP could be identified at the time-points and concentrations used in this study. This compound has low binding affinity to the AhR [20] and this may explain the lack of expression changes observed. No genes induced by BaP on the microarrays could therefore be eliminated from being potentially linked to carcinogenesis.

### DNA adducts and gene expression

In both MCF-7 and HepG2 cells, BaP-DNA adduct levels were similar, although time- and concentration effects were different (Figure 1). The recovery of gene expression identified in both cell lines is likely to be associated with levels of DNA damage. This indicates that concentration thresholds occur, below which gene expression levels return to normal after initial alteration. This threshold appears to be lower in the MCF-7 cells with recovery of gene expression occurring only after exposure to the lower concentrations of BaP (0.25 and 1 μM) (Figure 3). This reflects differences in the metabolic capabilities of the two cell lines to produce the ultimate carcinogenic metabolite of BaP, BPDE, and also suggests that HepG2 cells are more efficient at DNA repair.

A large number of the 27 gene expression changes common to both cell lines significantly correlated with DNA adduct levels in the MCF-7 cells (Table 5) indicating a direct link between their expression and DNA damage in this cell line. A number of these genes are likely to be directly linked to DNA adduct formation such as the xenobiotic metabolism genes whilst a number of expression changes are a direct result of the DNA damage, such as the p53-regulated gene *CDKN1A*. In the HepG2 cells fewer genes correlated well with DNA adduct levels suggesting that other mechanisms are regulating this response. If HepG2 cells are more efficient at detoxifying BaP and/or repairing the DNA damage, these processes could affect the relationship between DNA adduct levels and gene expression in this cell line. The time and concentration response of gene expression and DNA adduct formation coupled with the strong correlation of a number of genes with DNA adducts over time and concentration in both cell lines suggests that such genes could be useful biomarkers of the level of genotoxic compound exposure.

Although DNA adducts were detected in the cells after exposure to 0.25 μM BaP, few expression changes occurred in either cell line at this concentration. DNA adducts therefore still represent a more robust measure of carcinogen exposure at low dose levels. Similar results were observed by Akerman et al. [10] who analysed BPDE-induced gene expression changes and DNA adduct formation in TK6 cells.

### RTqPCR

RTqPCR was used to verify the microarray data for eight modulated genes and a good correlation between the two measures of expression was observed for both cell lines in the majority of cases (R > 0.634). An exception was *CYP1B1*, for which low correlations were observed for both the MCF-7 (R = 0.125) and HepG2 (R = -0.308) cells. Low correlation in the MCF-7 cells could be explained by its constant high induction across all time-points, measured by both the microarrays and RTqPCR. Pearson correlation looks for similar shapes in the two data-sets and in this case it is probably correlating on noise within the data. In HepG2 cells induction of *CYP1B1 *was observed only by RTqPCR and not by the micorarrays. *CYP1B1 *induction was also not observed in a microarray study by Staal et al. [9] who also analysed the effects of BaP exposure on gene expression in HepG2 cells. The failure of the microarrays to identify this gene expression change may be a result of very low basal levels of this transcript in this cell line, such that even if increased 40-fold, the microarrays are not sensitive enough to detect it. In this study *CYP1B1 *mRNA levels were much lower in HepG2 cells relative to MCF-7 cells as identified from the RTqPCR results. Overall RTqPCR analysis suggested that gene expression is underestimated on the microarrays, which may lead to some false negatives.

### Xenobiotic metabolism genes

A number of genes involved in xenobiotic metabolism and transcriptionally activated via the aryl hydrocarbon receptor (AHR) were up-regulated in both cell lines including *NQO1*, *AKR1C3*, *AKR1C1*, *MGST1 *and *CPM*. Detoxification of PAH quinone metabolites is carried out by the quinone oxidoreductase encoded by *NQO1 *[9,21], which is also required for p53 stabilisation in response to DNA damage [22]. The glutathione S-transferase *MGST1 *is also involved in cellular defence against toxic and carcinogenic electrophilic compounds by conjugation of reduced glutathione to hydrophobic electrophiles [23], so it is no surprise that this gene is up-regulated in response to BaP exposure. *AKR1C1 *and *AKR1C3 *both encode aldo-keto reductases capable of metabolising the PAH proximate carcinogens trans-dihydrodiols to *o*-quinones, which can lead to DNA damage directly through DNA adduct formation or indirectly through production of reactive oxygen species (ROS) [24]. Little is known about the function of the gene *CPM*, which encodes a carboxypeptidase; however, it is implicated in aromatic compound metabolism [25] and this may explain its up-regulation in response to BaP. As already discussed, *CYP1B1 *was strongly up-regulated by BaP in the MCF-7 cells at all time-points and concentrations on the microarrays but in HepG2 cells by RTqPCR analysis alone. This gene encodes a member of the cytochrome P450 superfamily of monooxygenases and is involved in the metabolic activation of PAHs including BaP [26,27]. Expression of this gene is inducible through the AHR for which BaP is a known ligand [28]. Enhanced expression of this protein has been observed in a number of cancers [29,30] and it has been demonstrated using CYP1B1-null mice that this enzyme enhances the carcinogenicity of the PAH 7,12-dimethylbenz(a)anthracene [31]. *CYP1B1 *has also been found to be consistently up-regulated in a panel of primary normal human mammary epithelial cells exposed to BaP [14] highlighting the importance of this enzyme in BaP metabolism in this tissue. Additional metabolising genes up-regulated in the HepG2 cells included *UGDH *and *GCLM*. *UGDH *encodes a glucose dehydrogenase needed for the glucuronidation reaction of xenobiotics that can decrease their mutagenicity [32]. *GCLM *encodes the modifier subunit of glutamate-cysteine ligase, an enzyme needed for glutathione synthesis, which is required for the detoxification of PAHs by enzymes such a MGST1 [33]. The greater number of detoxification genes induced in HepG2 cells implies that, as suggested above, this cell line is more efficient at detoxifying, as opposed to bioactivating, BaP than MCF-7 cells.

### Cell cycle regulation/proliferation genes

A number of genes involved in cell cycle regulation and proliferation were modulated in both cell lines, including *CDKN1A *and *MAK*. *CDKN1A *is a potent cell cycle inhibitor, regulating cell cycle progression at the G1 and G2 check-points in response to a variety of stress stimuli [34,35]. Transcriptional induction of this gene has been shown previously in human cells exposed to BaP [6,36]. The male germ cell-associated kinase *MAK *has been implicated in cell cycle regulation due to the similarity of its serine/threonine kinase protein product to kinases involved in cell cycle regulation [37]. In the MCF-7 cells, other up-regulated genes linked to cell cycle regulation included *BTG2*, *CCNG1*, *PA26*, and *SLC3A2 *and the down-regulated genes included *CCND1, AREG, BMP7, NET1, MYB*, and *IGFBP5*. *BTG2 *is also involved in G1 arrest of the cell cycle in response to DNA damage [38] and *CCNG1 *encodes cyclin G1, which has growth inhibitory activity [39]. Little is known, however, about the involvement of *PA26 *and *SLC3A2 *in cell cycle regulation. Down-regulated cell cycle genes in the MCF-7 cells included *CCND1*, which encodes the oncogene cyclin D1 whose expression is needed for G1 to S phase transition of the cell cycle [40]. This gene is frequently mutated, amplified or overexpressed in a number of different tumour types [40] and is likely to be involved in their pathogenesis [41]. Polymorphisms within this gene have been associated with lung cancer in a Chinese population study [42]. *AREG*, a member of the epidermal growth factor family, and *BMP7*, a bone morphogenic protein, are both linked to cell proliferation. These two genes were down-regulated in response to BaP along with *NET1*, which was originally cloned as a transforming gene in a screen for novel oncogenes in NIH 3T3 cells [43]. It was interesting to see the down-regulation of the protooncogene *MYB*. This gene encodes a transcription factor that controls differentiation and proliferation of a number of cell types [44] and has been linked to cell cycle control in haematopoietic progenitor cells [45]. Transcriptional down-regulation of this gene is another indication of cell-cycle arrest in response to BaP. Studies on the effect of *IGFBP5 *on cell growth suggest that its function as a promoter of growth inhibition or stimulation is cell-type specific [46,47], although a study by Butt et al. [48] demonstrated that in human breast cancer cells over-expression of this gene results in inhibition of cell growth and induction of apoptosis. Down-regulation of this gene cannot therefore be completely explained and may indicate that there are cell proliferation mechanisms acting against the cell-cycle arrest pathways. In the HepG2 cells other modulated genes involved in cell-cycle regulation and proliferation included *OKL38, IGFBP1, CTGF *and *FGG*. *OKL38*, the pregnancy-induced growth factor and *IGFBP1*, which has been shown to reduce the growth of prostate cancer cells in culture [49] were both induced in response to BaP. *FGG *and *CTGF *were both down-regulated in the HepG2 cells after BaP exposure. The down-regulation of *CTGF *in response to BaP was shown by Mahadevan et al. [6] in MCF-7 cells, although its repression in this cell line was not observed in this study. The involvement of *CTGF *and *FGG *in cell cycle regulation is not well understood, although there is direct evidence of the role of *CTGF *in the angiogenic mechanism [50] and *FGG *encodes the gamma component of fibrinogen which has also been shown to play a part in angiogenesis [51].

### Cell cycle analysis and gene expression

In both MCF-7 and HepG2 cells BaP exposure resulted in an arrest of the cells in S phase of the cell cycle (Table 7), indicative of DNA synthesis being interrupted. This is in agreement with other studies that have shown the inhibition of DNA synthesis in response to BaP [52,53]. This pause in DNA synthesis is probably to allow repair enzymes to recognize the damaged DNA and correct it [54]. Interestingly, in both cell lines a number of histone genes were down-regulated, the synthesis of which is tightly coupled with DNA synthesis during S phase of the cell cycle [55] and their down-regulation has been seen in response to ionising radiation-induced DNA damage [55]. It is therefore likely that down-regulation of histone genes in response to BaP is linked to DNA synthesis inhibition in these two cell lines. These genes showed significant negative correlation with DNA adduct levels in the MCF-7 cells but this was not the case for the HepG2 cells. The increasing down-regulation of the histone genes in MCF-7 cells exposed to BaP for 24 and 48 h is coupled to the arrest of these cells in S phase at 24 and 48 h. In HepG2 cells, however, this association was not so clearly observed. After down-regulation at 6 h, these genes were then up-regulated at 24 h and then their expression returned to normal after 48 h, whereas arrest of the HepG2 cells in S phase was observed at both 24 and 48 h. The initial repression followed by induction of these genes in the HepG2 cells indicates that DNA synthesis is only temporarily inhibited in HepG2 cells, again suggesting that these cells are better at repairing the DNA damage.

Over-represented biological processes in the data included G2/M regulation in both cell lines and G1/S regulation also in the MCF-7 cells through induction of cell cycle inhibitor genes such as *CDKN1A*. Although there was no strong accumulation of the cells in G1 or G2 in response to BaP it may be that these effects are seen at later time-points than those analysed here as indicated by the minor effects seen at 72 h.

### p53-regulated genes

In both cell lines, the expression of genes that are known direct targets of the tumour suppressor protein p53 were induced, including *CDKN1A, BTG2, PA26, CCNG1, BAX *and *DDB2 *in the MCF-7 cells and *CDKN1A *and *BAX *in the HepG2 cells. The larger p53 response in the MCF-7 cells (Figure 7) again reflects the higher DNA adduct levels detected in this cell line. Increased cellular p53 protein levels in response to various genotoxic agents are due mainly to an increase in p53 protein stability by post-translational modification rather than an increase in steady-state p53 mRNA levels [56] and this would explain why up-regulation of this transcript was not observed on the microarrays. Comparison of the p53 protein levels with gene expression profiles of p53-regulated genes showed that protein and gene induction occurred in a similar manner (Figure 7 and 8).

### Apoptosis/anti-apoptosis genes

In addition to a cell cycle response to BaP, apoptotic signalling was also evident in the cells. Induction of the pro-apoptotic gene,*BAX*, in MCF-7 cells by BaP was shown by microarray analysis and by RTqPCR. In the HepG2 cells induction of this gene was detected by RTqPCR but was not identified as a significant expression change on the microarrays. This gene is regulated by p53 and its increased expression in response to BaP has been shown previously [3]. The immediate early response gene *IER3 *was also up-regulated by BaP in both cell lines. The protein encoded by this gene can be either an inducer or inhibitor of apoptosis [57-59]. Enhanced expression of this gene may therefore result in deficient apoptosis, which may contribute to malignant transformation by increased cell survival allowing for tumour promotion and progression. Further studies, however, are needed to determine the apoptotic function of this gene in response to BaP. Another gene linked to anti-apoptotic signalling is *BIRC5 *(survivin), a member of the inhibitor of apoptosis (IAP) gene family, and this was up-regulated in HepG2 cells. This gene prevents apoptotic cell death [60] and is over-expressed in a number of different cancers [61]. It is interesting that although apoptotic signalling is occurring which would prevent mutagenesis, anti-apoptotic signalling also occurs in response to BaP that potentially enhances survival of mutated cells [62].

### DNA repair genes

There was no great response in nucleotide excision repair genes on the microarrays in response to BaP and this is consistent with other microarray studies that have looked at the effects of BaP or its metabolites [3,8,10]. This is likely to be due to low constitutive expression of these important defence genes. Exceptions included the p53-regulated *DDB2 *gene, which encodes a DNA-damage recognition protein needed for NER [63,64] and whose expression was enhanced in the MCF-7 cells but not HepG2. *CRY1*, modulated by BaP in both cell lines, encodes an enzyme implicated in the repair of cyclobutyl pyrimidine dimers, although its exact function is not fully understood and its relevance in the BaP response is not immediately apparent. *PCNA*, which encodes a protein involved in both replication and repair [65], was up-regulated in the HepG2 cells after exposure to 5 μM BaP but not in MCF-7 cells.

### Other genes and effects

Other interesting expression changes that were common to both cell lines included the down-regulation of *SCD*. Loss of expression of this stearoyl-CoA desaturase enzyme, which is involved in fatty acid metabolism, is a frequent event in prostate carcinoma [66]. *GDF15*, implicated in signal transduction, was strongly and consistently induced in both cell lines. Transcription of this member of the TGF-β superfamily can be p53-dependent or -independent and has been shown to have anti-tumourigenic and pro-apoptotic activity in cells derived from a human colorectal adenocarcinoma [67]. Interestingly, this gene has been identified as up-regulated in a number of different cancer types [68]. Evidence of oxidative damage within the cells could also be seen from the BaP expression patterns. *TXNRD1 *is involved in protecting cells from oxidative stress [69,70], indicating the generation of reactive oxygen species after BaP exposure, which may contribute to the DNA damage burden caused by this compound.

## Conclusion

This study has shown alterations of gene expression in human cells treated with BaP at concentrations at which DNA adduct formation occurs, with minimal cytotoxicity, giving greater insight into the cellular response to carcinogen exposure. By linking gene expression data to other phenotypic measures, such as DNA damage levels, cell cycle analysis, and p53 protein expression, we have further elucidated the roles of environment and gene interactions, which may be important in the multi-step process of carcinogenesis. Overall, the response consisted of up-regulation of tumour suppressor genes and down-regulation of oncogenes promoting cell cycle arrest and apoptosis that would aid in protecting the cells from mutagenic transformation by the carcinogen. Anti-apoptotic signalling was also identified, however, which may increase cell survival and promote tumourigenesis. A number of the genes identified have been induced in normal human cells by BaP (e.g. *CYP1B1 *and *NQO1*) [14] and this gives promise that the expression changes we are observing in these two cell systems are not likely to be artefacts of their cancer phenotype. As tumour cell lines have been used in this study it will be necessary to extend the analysis to primary cells to refine the determination of the critical events in carcinogen-induced gene regulation. Investigations are in progress to further characterise the BaP-expression response and to determine which BaP-expression changes result from AHR-activation and which result from DNA damage.

## Methods

### Cell culture and chemical treatment

MCF-7 human breast carcinoma cells and HepG2 human hepatocarcinoma cells were purchased from the European Collection of Cell Cultures (ECACC, Salisbury, UK). The cells were grown as adherent monolayers and maintained in Eagle's MEM supplemented with 2 mM L-glutamine, 1 mM sodium pyruvate, 1% non-essential amino acids, 10% foetal bovine serum, 100 U penicillin/ml and 100 μg streptomycin/ml. All media were purchased from Sigma Aldrich, UK. Sub-culturing was performed every 72 h when the cells were 80% confluent and incubated in a humidified 5% CO_2 _atmosphere at 37°C. BaP and BeP were both obtained from Sigma Aldrich. For chemical exposure, T75 flasks were seeded at 0.13 × 10^6 ^MCF-7 cells/ml or 0.27 × 10^6 ^HepG2 cells/ml in a total volume of 15 ml and after 48 h the appropriate concentrations of BaP or BeP dissolved in DMSO (Sigma Aldrich) were added. DMSO alone was added to control cultures and its volume was kept at 0.2% of the total culture medium. All cell incubations for the different experimental applications were carried out in duplicate and cells were harvested by trypsinisation and then washed with PBS.

### Cell viability and DNA adduct measurement

Cells were exposed to BaP (0.01, 0.10, 0.25, 0.50, 1.00, 2.50, or 5.00 μM), BeP (2.50 or 5.00 μM) or DMSO alone and cell viability and DNA adducts were measured. Cell viability was measured using a Vi-Cell Cell Viability Analyzer (Beckman Coulter, UK) after exposure to the compounds for up to 96 h. Numbers of DMSO-exposed control cells were taken to be 100% viable and from this, percentage viability was calculated for exposed cells. Cells were spun down, and from each pellet DNA was isolated by a standard phenol chloroform extraction method and DNA adducts were measured for each DNA sample using the nuclease P1 enrichment version of the ^32^P-postlabelling method as described previously with minor modifications [71] after exposure of the compounds for up to 48 h. Solvent conditions for the resolution of ^32^P-labelled adducts on polyethyleneimine-cellulose TLC were: D1, 1.0 M sodium phosphate, pH 6.0; D3, 3.5 M lithium formate, 8.5 M urea, pH 3.5; D4, 0.8 M lithium chloride, 0.5 M Tris, 8.5 M urea, pH 8.0. DNA adduct levels (RAL, relative adduct labelling) were calculated from the adduct cpm, the specific activity of [γ-^32^P]ATP and the amount of DNA (pmol of DNA-P) used. Results were expressed as DNA adducts/10^8 ^nucleotides. An external BPDE-DNA standard [72] was employed for identification of adducts in experimental samples.

### RNA extraction and cDNA synthesis for microarray analysis

Cells were treated with 0.25, 1.00, 2.50 or 5.00 μM BaP or DMSO as vehicle control. MCF-7 cells were also treated with equimolar concentrations (0.25 – 2.5 μM) of BeP and HepG2 cells were treated with 5.00 μM BeP. Cells were exposed for 6, 24 and 48 h, cell pellets collected and total RNA extracted using the Qiagen RNeasy Mini Kit protocol (RNeasy Mini Handbook, Qiagen, UK). After addition of lysis buffer (RLT, Qiagen) samples were homogenized using QIAshredders (Qiagen). RNA was quantified spectrophotometrically, and integrity was determined using a 2100 Bioanalyzer (Agilent Technologies, UK). RNA that had an rRNA 28S/18S ratio >1.5 was used for microarray analysis to achieve optimal labelling with the fluorophores.

Total RNA (4 μg) was reversed transcribed into cDNA and fluorescently labelled with Cy3 or Cy5 mono-reactive dyes (Amersham Biosciences, UK) using the Invitrogen Indirect cDNA Labelling Kit protocol (Invitrogen, UK) according to the manufacturer's instructions. After labelling, repetitive sequences within the cDNA samples were blocked with 16 μg Human Cot-1 DNA (Invitrogen, UK) to prevent non-specific sequences binding to the cDNA probes.

### cDNA microarray hybridisations

Gene expression analysis was carried out using the Cancer Research UK DNA Microarray Facility (CRUKDMF) Human 15 K Genome-Wide Array v 1.1.0 and the Human 6 K Genome-wide Array v1.0.0. Full probe lists for these arrays can be found at the CRUKDMF Microarray Publications website [73]. Only the 2.5 and 5 μM BaP-treated samples were analysed on the 6 K array. The majority of the clones have been sequence verified and are 800–2,000 bp in length. The arrays were gridded onto either poly-L-lysine coated slides or Type 7* silanised slides (GE Healthcare, UK). To prepare the poly-L-lysine slides, Gold Seal glass slides (Merck Eurolab Ltd, Poole, UK) were washed and coated with poly-L-lysine (Invitrogen, UK) and after gridding free poly-L-lysine molecules were blocked, all as previously described [74]. The gridded poly-L-lysine slides were washed prior to hybridisation by denaturing in 70% deionised formamide/2X SSC pH 7.0 at 65°C for 2 min and then washed successively with 70% ethanol twice, 80% ethanol and 100% ethanol, blow-dried with nitrogen gas and pre-warmed in a hybridisation chamber at 65°C. The Type 7* slides did not require preparation or pre-hybridisation washing and after gridding and UV cross-linking (1,000 mJ), they were ready for pre-warming in the hybridisation chamber.

Labelled sample was reduced down to 3 μl using Microcon YM-30 centifugal filters (Millipore, UK). Corresponding control and treated samples labelled with different fluorophores were combined in a 50 μl hybridisation mix (19 μl Microarray Hybridisation Solution purchased from GE Healthcare and 50% deionised formamide) and heated at 70°C for 2 min and then at 37°C for 10 min. Sample was pipetted onto a microarray slide and covered with a glass cover slip. A 150μl aliquot of 6 X SSPE was pipetted underneath the slide to prevent dehydration. The hybridisation chamber was sealed and incubated at 42°C for 72 h. Poly-L-lysine slides were given three washes in 4 X SSPE, 10 mM EDTA pH 8.0 at 42°C. The first was a brief soak, the second was to remove the coverslip and the third was performed for 1 min. These slides were then washed in 2 X SSPE, 10 mM EDTA pH 8.0 for 15 s at RT, and then in 0.1 X SSPE for 15 s at RT before briefly rinsing in HPLC grade water and drying with nitrogen gas. Type 7* slides were washed three times in 4 X SSPE, 10 mM EDTA pH 8.0 as above, followed by a 10 s wash in 50% deionised formamide, 6 X SSPE, before a brief rinse in HPLC grade water and drying with nitrogen gas. The slides were then scanned on an Axon Genepix 4000A laser scanner (Axon Instruments, USA).

### Microarray analysis

The initial analysis of the scanned slides was carried out using GenePix Pro v-5.1 software (Axon Instruments, USA). Within GenePix software the quality of individual spots was assessed. A spot was automatically flagged to be included in normalization (present) if the signal intensity of >75% of the pixels for either the Cy3 and Cy5 channels were 1S.D. above the background intensity. In addition, spot quality was also assessed visually. Background subtraction is not suitable for these microarrays and in preliminary analysis normalisation of background-corrected data was shown to decrease the data quality as compared to normalisation without background correction. Raw data generated from GenePix software were therefore imported into GeneSpring v-7.2 software (Agilent Technoligies, UK) without background subtraction and subjected to print-tip Lowess normalisation [75] to allow different arrays to be compared. Within GeneSpring, genes were removed from further analysis if they had not been flagged in Genepix software as present in greater than 50% of the experimental samples. As multiple time and concentration sample points were used for these analyses, biological replicates but not technical replicates were performed so that the microarrays could be utilized for the additional sampling points. Cy3/Cy5 ratios of the biological replicate samples were averaged and these data were then used to identify modulated genes within the data using a fold-change cut-off of 1.4. Within Genespring a one-sample Student's T-test is calculated for replicate data to test whether the mean normalized expression value for the gene is statistically different from 1. Log2 transformed averaged Cy3/Cy5 ratios were used to identify modulated genes that had a one-sample Student's T-test *p*-value assigned by GeneSpring of less than 0.05. Log2 transformed data were used for any statistical algorithms performed within GeneSpring such as 2-Way ANOVA and PCA. Pearson Correlation coefficients of gene expression data and DNA adduct data were calculated using Microsoft Excel.

The gene expression data discussed in this publication have been deposited in NCBI Gene Expression Omnibus [76] and are accessible through GEO Series accession number GSE5894.

### Real-time quantitative PCR

Two-step reverse transcription-PCR was used to generate cDNA for relative quantitation analysis using real-time fluorescent PCR. The cDNAs were reversed transcribed from 1 μg total RNA using random primers and following the Superscript III Reverse Transcriptase First-Strand cDNA Synthesis Protocol (Invitrogen, UK). The cDNA was diluted 1:10 and 2μl was used as template to perform RT-PCR in a 15μl reaction. *GAPDH *was used as an endogenous control (Applied Biosystems, UK) in multiplexed PCR reactions on an ABI PRISM 7900HT Sequence Detection System (Applied Biosystems) with standard thermocycling conditions (50°C 2 min, 95°C 10 min, then 40 cycles of 95°C 15 s, 60°C 1 min), using Taqman Universal PCR Master Mix (Applied Biosystems). To confirm the modulated expression of the selected target genes 20x Assays-On-Demand™ gene expression primers and probes (Applied Biosystems) were used (CYP1B1-Hs00164383_m1, NQO1-Hs00168547_m1, AKR1C3-Hs00366267_m1, CYP1A1-Hs00153120_m1, Bax-Hs00180269, PCNA-Hs00427214_g1, TP53-Hs00153349_m1, AhR-Hs00169233_m1, CDKN1A-Hs00355782_m1, HIST1H3D-Hs00371415_m1, IER3-Hs00174674_m1). All PCR reactions were performed in triplicate and changes in gene expression between the control (or calibrator) and treated samples after normalisation to the *GAPDH *reference were calculated using the comparative threshold cycle (C_T_) method where relative amount = 2−ΔΔCT
 MathType@MTEF@5@5@+=feaafiart1ev1aaatCvAUfKttLearuWrP9MDH5MBPbIqV92AaeXatLxBI9gBaebbnrfifHhDYfgasaacH8akY=wiFfYdH8Gipec8Eeeu0xXdbba9frFj0=OqFfea0dXdd9vqai=hGuQ8kuc9pgc9s8qqaq=dirpe0xb9q8qiLsFr0=vr0=vr0dc8meaabaqaciaacaGaaeqabaqabeGadaaakeaacqaIYaGmdaahaaWcbeqaaiabgkHiTiabfs5aejabfs5aejabboeadnaaBaaameaacqqGubavaeqaaaaaaaa@33ED@, and where ΔΔC_T _is the ΔC_T _of the target gene (threshold cycle test gene – threshold cycle endogenous control) minus the ΔC_T _of the calibrator sample (threshold cycle calibrator gene – threshold cycle endogenous control).

### Western blot analysis

Cells were exposed to BaP (1.00 or 5.00 μM), BeP (5.00 μM) or DMSO alone for up to 48 h. Cells were trypsinised and pellets collected after 6, 24 and 48 h exposure and then lysed in 100 μl lysis buffer (50 mM HEPES pH 7.4, 250 mM NaCl, 0.1% NP40, 1 mM DTT, plus 1 tablet of protease inhibitor cocktail from Roche, Lewes, UK) on ice for 30 min. Cell lysates were electrophoretically separated using NuPage 4–12% Bis-Tris SDS polyacrylamide gels (Invitrogen, UK). Following electrophoresis, gels were transferred onto an Immunobilon-P PVDF membrane (Millipore, UK). The membranes were then blocked by incubation in 5% non-fat dry milk in tris buffered saline for 1 h at room temperature followed by a second blocking step using 10% milk for 15 min. The blots were then incubated with primary antibody and then with the species-specific horseradish peroxidase-conjugated secondary antibody (Bio-Rad) and bands detected by chemiluminescence (ECL detection reagents, GE Healthcare). The monoclonal antibody against p53 (Ab-6) was purchased from Calbiochem (Darmstadt, Germany) and diluted 1:5000. Monoclonal antibody to detect β-actin (Ab8226) was purchased from Abcam (Cambridge, UK), diluted 1:2000 and used as a loading control.

### Flow cytometry

Cells were exposed to 2.50 μM BaP or DMSO alone and harvested by trypsinisation after 24, 48 and 72 h. The cell pellets were re-suspended in 0.2 ml 10 X PBS solution and fixed in 2 ml of ice-cold 70% ethanol. The samples were then stored at 4°C for at least 30 min prior to use. Twenty-four hours prior to flow cytometry analysis the samples were centrifuged at 1500 × g for 5 min and resuspended in staining buffer containing 40 μg/ml propidium iodide (Molecular Probes, Invitrogen, UK), 100 μg/ml RNase (Sigma Aldrich, UK) in PBS buffer so that final concentration was equal to 1 × 10^6 ^cells/ml. The cells were then incubated at 37°C for 30 min and then returned to 4°C overnight. Cell cycle analysis was performed using a Beckman Coulter EPICS Elite ESP (Beckman Coulter, Buckinghamshire, UK) at 488 nm. The relative number of cells in each phase of the cell cycle was determined using Cylcred v 1.0.2 and WinMidi v2.8 software [77].

## Authors' contributions

DP conceived the study and supervised its design and coordination. SH, IG and VA participated in design and coordination of the study. IG designed and manufactured the microarrays. SH carried out all experiments with the exception of DNA adduct measurements which were performed by VA. Data analysis was performed by SH and DB. SH drafted the manuscript and DP and IG participated in its preparation. All authors have read and approved the final manuscript.

## Supplementary Material

Additional file 1 MCF-7 and HepG2 BaP expression changes by 1.4-fold. The data presented in each sheet are genes with an expression ratio of > 1.4 or < 0.714 (i.e. genes with a 1.4-fold change) in at least one sample and had a Student's one-sample T-test p-value of < 0.05 in at least one sample after benzo(a)pyrene exposure. Data from the 6 K and 15 K microarrays are given for each cell line. Click here for file

Additional file 2 HepG2 2-Way ANOVA gene lists. The table lists genes identified in HePG2 cells by 2-way ANOVA as being significantly (p < 0.05) affected by BaP concentration and exposure time. Click here for file

Additional file 3 MCF-7 2-Way ANOVA gene lists. The table lists genes identified in MCF-7 cells by 2-way ANOVA as being significantly (p < 0.05) affected by BaP concentration and exposure time. Click here for file

Additional file 4 MCF-7 and HepG2 BeP expression changes by 1.4-fold. The data presented in each sheet are genes with an expression ratio of > 1.4 or < 0.714 (i.e. genes with a 1.4-fold change) in at least one sample and had a p-value of < 0.05 in at least one sample after benzo(e)pyrene exposure. Click here for file

Additional file 5 EASE results. The data presented are biological processes significantly (fisher exact probability p < 0.05) over-represented in the MCF-7 and HepG2 time and concentration BaP-modulated gene lists as calculated by EASE. Click here for file

Additional file 6 RTqPCR data. Differential expression of selected genes in MCF-7 and HepG2 cells exposed to BaP measured by microarray and RTqPCR. Click here for file

## Figures and Tables

**Table 1:** Numbers of genes identified by 2-Way ANOVA from the 15 K and 6 K microarray data as being differentially expressed as an effect of BaP or BeP exposure time or concentration in MCF-7 and HepG2 cells

2-Way ANOVA Parameters	Number of gene expression changes			
	MCF-7		HepG2	
	BaP	BeP	BaP	BeP
Time	130	12	68	0
Concentration	74	2	55	0
Time and concentration overlap	67	1	39	0

**Table 2:** Biological processes significantly affected (Fisher exact test p value < 0.05) by BaP in MCF-7 cells as determined by EASE

**MCF-7 time and concentration down-regulated genes**		**MCF-7 time and concentration up-regulated genes**	
**Biological Process**	**Probability**	**Biological Process**	**Probability**
regulation of actin filament length	4.9E-06	water-soluble vitamin metabolism	3.7E-03
actin cytoskeleton organization and biogenesis	1.8E-04	sodium\:calcium exchange	6.2E-03
actin filament-based process	2.4E-04	pentose-phosphate shunt, oxidative branch	6.2E-03
regulation of actin polymerization and/or depolymerization	4.0E-04	glyoxylate cycle	6.2E-03
one-carbon compound metabolism	4.6E-04	glyoxylate metabolism	6.2E-03
cell organization and biogenesis	8.2E-04	vitamin metabolism	8.6E-03
nucleosome assembly	9.0E-04	amino acid transport	1.2E-02
carboxylic acid metabolism	1.5E-03	thiamin and derivative metabolism	1.2E-02
organic acid metabolism	1.6E-03	embryonic eye morphogenesis	1.2E-02
actin polymerization and/or depolymerization	2.2E-03	solute, solute exchange	1.2E-02
amine metabolism	2.4E-03	thiamin metabolism	1.2E-02
establishment and/or maintenance of chromatin architecture	3.2E-03	internal protein amino acid acetylation	1.2E-02
DNA packaging	4.3E-03	eye morphogenesis (sensu Vertebrata)	1.2E-02
chromatin assembly/disassembly	4.7E-03	eye morphogenesis (sensu Mammalia)	1.2E-02
amino acid metabolism	4.7E-03	isocitrate metabolism	1.2E-02
cytoskeleton organization and biogenesis	5.8E-03	amine/polyamine transport	1.3E-02
chromosome organization and biogenesis (sensu Eukarya)	6.4E-03	regulation of CDK activity	1.4E-02
muscle maintenance	6.5E-03	negative regulation of cell proliferation	1.6E-02
negative regulation of actin filament polymerization	6.5E-03	GPI anchor biosynthesis	1.8E-02
anion homeostasis	6.5E-03	GPI anchor metabolism	1.8E-02
monovalent inorganic anion homeostasis	6.5E-03	regulation of survival gene product activity	1.8E-02
asparagine metabolism	6.5E-03	negative regulation of survival gene product activity	1.8E-02
sequestering of actin monomers	6.5E-03	G2/M transition of mitotic cell cycle	1.9E-02
asparagine biosynthesis	6.5E-03	carboxylic acid transport	2.1E-02
nuclear organization and biogenesis	6.8E-03	organic acid transport	2.1E-02
amino acid and derivative metabolism	7.8E-03	apoptotic mitochondrial changes	2.4E-02
cell growth	1.0E-02	germ-cell development	2.4E-02
myelination	1.3E-02	protein amino acid acetylation	2.4E-02
isoleucyl-tRNA aminoacylation	1.3E-02	G1/S transition of mitotic cell cycle	2.5E-02
threonyl-tRNA aminoacylation	1.3E-02	NADPH metabolism	3.0E-02
regulation of actin filament polymerization	1.3E-02	NADPH regeneration	3.0E-02
organelle organization and biogenesis	1.7E-02	CTP biosynthesis	3.0E-02
salivary gland morphogenesis	1.9E-02	UTP metabolism	3.0E-02
salivary gland development	1.9E-02	UTP biosynthesis	3.0E-02
folic acid and derivative biosynthesis	2.6E-02	pentose-phosphate shunt	3.0E-02
glycine metabolism	2.6E-02	CTP metabolism	3.0E-02
cytoplasm organization and biogenesis	2.6E-02	synaptic transmission\, cholinergic	3.0E-02
actin filament polymerization	3.2E-02	eye morphogenesis	3.0E-02
folic acid and derivative metabolism	3.2E-02	pyrimidine ribonucleoside triphosphate metabolism	3.0E-02
regulation of cell growth	3.8E-02	pyrimidine ribonucleoside triphosphate biosynthesis	3.0E-02
aspartate family amino acid biosynthesis	3.8E-02	pyrimidine ribonucleotide biosynthesis	3.6E-02
L-serine metabolism	3.8E-02	GTP metabolism	3.6E-02
nerve ensheathment	4.4E-02	GTP biosynthesis	3.6E-02
negative regulation of cell adhesion	4.4E-02	pyrimidine ribonucleotide metabolism	3.6E-02
nerve maturation	4.4E-02	nicotinamide metabolism	3.6E-02
glycosphingolipid biosynthesis	4.4E-02	pyrimidine nucleoside triphosphate metabolism	3.6E-02
tRNA aminoacylation for protein translation	4.6E-02	phosphoinositide biosynthesis	3.6E-02
tRNA aminoacylation	4.6E-02	oncogenesis	3.8E-02
amino acid activation	4.6E-02	DNA repair	4.2E-02
tRNA modification	4.7E-02	covalent chromatin modification	4.2E-02
		phosphoinositide metabolism	4.2E-02
		histone deacetylation	4.2E-02
		pyridine nucleotide metabolism	4.2E-02
		heavy metal sensitivity/resistance	4.2E-02
		nerve-nerve synaptic transmission	4.2E-02
		histone modification	4.2E-02
		aldehyde metabolism	4.2E-02
		protein modification	4.3E-02
		main pathways of carbohydrate metabolism	4.5E-02
		obsolete biological process	4.7E-02
		prostanoid metabolism	4.8E-02
		embryonic development (sensu Vertebrata)	4.8E-02
		lipoprotein biosynthesis	4.8E-02
		insulin receptor signaling pathway	4.8E-02
		prostaglandin metabolism	4.8E-02
		embryonic development (sensu Mammalia)	4.8E-02
		protein lipidation	4.8E-02

**Table 3:** Biological processes significantly affected (Fisher exact test p value < 0.05) by BaP in HepG2 cells as determined by EASE

**HepG2 time and concentration down-regulated genes**		**HepG2 time and concentration up-regulated genes**	
**Biological Process**	**Probability**	**Biological Procees**	**Probability**
nucleosome assembly	2.0E-04	nucleosome assembly	4.5E-04
establishment and/or maintenance of chromatin architecture	4.7E-04	prostaglandin metabolism	6.9E-04
DNA packaging	6.4E-04	prostanoid metabolism	6.9E-04
chromosome organization and biogenesis (sensu Eukarya)	9.7E-04	establishment and/or maintenance of chromatin architecture	1.3E-03
nuclear organization and biogenesis	1.0E-03	DNA packaging	1.8E-03
chromatin assembly/disassembly	1.1E-03	chromatin assembly/disassembly	2.4E-03
complement activation, classical pathway	4.5E-03	chromosome organization and biogenesis (sensu Eukarya)	2.7E-03
complement activation	6.0E-03	nuclear organization and biogenesis	2.9E-03
vacuolar protein catabolism	7.8E-03	icosanoid metabolism	3.3E-03
DNA metabolism	9.5E-03	blood coagulation	3.6E-03
blood coagulation	2.3E-02	hemostasis	4.5E-03
transcription from Pol II promoter	2.7E-02	glucuronate metabolism	5.1E-03
hemostasis	2.7E-02	uronic acid metabolism	5.1E-03
morphogenesis	3.6E-02	UDP-glucuronate biosynthesis	5.1E-03
humoral defense mechanism (sensu Vertebrata)	3.7E-02	glucuronate biosynthesis	5.1E-03
FGF receptor signaling pathway	3.8E-02	UDP-glucuronate metabolism	5.1E-03
skeletal development	4.7E-02	obsolete biological process	5.3E-03
		DNA metabolism	7.1E-03
		xenobiotic metabolism	9.4E-03
		response to xenobiotic stimulus	1.0E-02
		keratinocyte differentiation	1.0E-02
		endothelial cell differentiation	1.0E-02
		UDP-glucose metabolism	1.0E-02
		myoblast differentiation	1.0E-02
		cell growth and/or maintenance	1.1E-02
		G2/M transition of mitotic cell cycle	1.4E-02
		cellular process	1.4E-02
		cell organization and biogenesis	1.4E-02
		lipid transport	1.5E-02
		cysteine metabolism	1.5E-02
		embryogenesis and morphogenesis	2.3E-02
		synaptic transmission, cholinergic	2.5E-02
		actin filament polymerization	2.5E-02
		oncogenesis	2.7E-02
		response to chemical substance	2.9E-02
		electron transport	2.9E-02
		regulation of cell migration	3.0E-02
		glycosaminoglycan biosynthesis	3.0E-02
		regulation of DNA replication	3.5E-02
		N signaling pathway	3.5E-02
		heavy metal sensitivity/resistance	3.5E-02
		nerve-nerve synaptic transmission	3.5E-02
		aminoglycan biosynthesis	3.5E-02
		glutathione biosynthesis	3.5E-02
		regulation of apoptosis	4.0E-02
		glutathione metabolism	4.0E-02
		nucleotide-sugar metabolism	4.0E-02
		negative regulation of apoptosis	4.5E-02
		anti-apoptosis	4.5E-02
		carboxylic acid metabolism	4.7E-02
		organic acid metabolism	4.8E-02
		lipoprotein metabolism	5.0E-02
		nitric oxide metabolism	5.0E-02
		nitric oxide biosynthesis	5.0E-02

**Table 4:** Biological processes significantly affected by BaP in both MCF-7 and HepG2 cells

**Time and concentration down-regulated genes**	**Time and concentration up-regulated genes**
nucleosome assembly	G2/M transition of mitotic cell cycle
establishment and or maintenance of chromatin architecture	heavy metal sensitivity/resistance
DNA packaging	nerve-nerve synaptic transmission
chromosome organization and biogenesis (sensu Eukarya)	obsolete biological process
nuclear organization and biogenesis	oncogenesis
chromatin assembly/disassembly	prostaglandin metabolism
	prostanoid metabolism
	synaptic transmission, cholinergic

**Table 5:** Genes that were significantly altered by at least 1.4-fold in response to BaP in both MCF-7 and HepG2 cells

**IMAGE Clone ID**	**Gene Symbol**	**Genbank Accession Number**	**Gene Title**	**Biological Process**	**Direction of expression alteration^2^**		**Pearson correlation with DNA adducts**	
					**MCF-7**	**HepG2**	**MCF-7**	**HepG2**
1473304	AKR1C3	BQ939577	Aldo-keto reductase family 1, member C3 (3-alpha hydroxysteroid dehydrogenase, type II)	cell proliferation; prostaglandin metabolism	↑	↑	0.261	-0.065
810724	IER3	BM994398	Immediate early response 3	anti-apoptosis; cell growth and/or maintenance; embryogenesis and morphogenesis; morphogenesis	↑	↑	0.192	**0.805**^1^
789376	TXNRD1	NM_003330	Thioredoxin reductase 1	electron transport; heavy metal sensitivity/resistance; signal transduction	↑	↑	**0.849**	0.253
768443	MGST1	AK123482	Microsomal glutathione S-transferase 1	prostaglandin metabolism	↑	↑	**0.599**	**0.815**
196992	AKR1C1	AK095239	Aldo-keto reductase family 1, member C1 (dihydrodiol dehydrogenase 1; 20-alpha (3-alpha)-hydroxysteroid dehydrogenase)	xenobiotic metabolism	↑	↑	0.383	0.078
214006	HIST1H2BG	NM_003518	Histone 1, H2bg	nucleosome assembly; regulation of transcription\, DNA-dependent	↓	↓↑	**-0.780**	-0.201
1842170	HIST1H4B	BX102654	Histone 1, H4b	nucleosome assembly; regulation of transcription\, DNA-dependent	↓	↓↑	**-0.620**	-0.173
129467	HIST1H2BJ	BF983642	Histone 1, H2bj	nucleosome assembly; regulation of transcription\, DNA-dependent	↓	↓↑	**-0.709**	-0.193
268891	HIST1H3D	BQ051491	Histone 1, H3d	embryogenesis and morphogenesis; nucleosome assembly	↓	↓↑	**-0.896**	-0.214
263087	CRY1	AK125915	Cryptochrome 1 (photolyase-like)	DNA repair; circadian rhythm; vision	↑	↑	**0.805**	0.215
140997	GDF15	BQ883534	Growth differentiation factor 15	signal transduction	↑	↑↓	**0.940**	**0.716**
273392	PIGF	BM558246	Phosphatidylinositol glycan, class F	GPI anchor biosynthesis	↑	↑	**0.908**	**0.847**
376772	RPS27L	BC047648	Ribosomal protein S27-like	cell proliferation; protein biosynthesis; signal transduction	↑	↑	**0.944**	0.380
1461138	HIST1H4C	CD048335	Histone 1, H4c	nucleosome assembly; regulation of transcription\, DNA-dependent	↓	↓↑	**-0.706**	-0.156
205819	CPM	NM_001874	Carboxypeptidase M	aromatic compound metabolism; morphogenesis; proteolysis and peptidolysis	↑	↑	**0.930**	**0.768**
1673711	GOLGA1	U51587	Golgi autoantigen, golgin subfamily a, 1		↑↓	↑	-0.194	-0.206
166273	CDKN1A	NM_078467	Cyclin-dependent kinase inhibitor 1A (p21, Cip1)	cell cycle arrest; induction of apoptosis by intracellular signals; negative regulation of cell proliferation; oncogenesis; regulation of CDK activity	↑	↑	**0.947**	**0.713**
324356	NQO1	NM_000903	NAD(P)H dehydrogenase, quinone 1	electron transport; nitric oxide biosynthesis; response to toxin; synaptic transmission\, cholinergic; xenobiotic metabolism	↑	↑	**0.884**	-0.023
49442	TXNRD1	NM_003330	Thioredoxin reductase 1	electron transport; heavy metal sensitivity/resistance; signal transduction	↑	↑	**0.866**	0.003
233273	STOM	NM_004099	Stomatin		↑	↑	**0.925**	**0.886**
25398	UNC84A	BX640866	Unc-84 homolog A (C. elegans)		↓	↓↑	**-0.662**	-0.097
731750	TAF6L	BC008785	TAF6-like RNA polymerase II, p300/CBP-associated factor (PCAF)-associated factor, 65kDa	chromatin remodeling; regulation of transcription from Pol II promoter; transcription initiation	↓	↓↑	**-0.802**	-0.210
381786	MAK	NM_005906	Male germ cell-associated kinase	protein amino acid phosphorylation; spermatogenesis	↑	↑	**0.869**	0.493
323511	C7ORF36	BX648555	Chromosome 7 open reading frame 36		↑	↑	**0.917**	**0.567**
760246	BLOC1S2	NM_001001342	Biogenesis of lysosome-related organelles complex-1, subunit 2		↑	↑	**0.971**	**0.886**
233279	SCD	NM_005063	Stearoyl-CoA desaturase (delta-9-desaturase)	fatty acid biosynthesis	↓	↓	-0.372	-0.482
24032	CRADD	AL832705	CASP2 and RIPK1 domain containing adaptor with death domain	induction of apoptosis via death domain receptors; signal transduction	↑	↓	0.359	0.420

^1^Values in bold are significant Pearson correlations (p < 0.05).

^2^Genes with arrows in both directions represent those that were down-regulated at one time-point and then up-regulated at another or vice versa.

**Table 6:** Pearson correlation analysis of selected gene expression changes measured by microarray and RTqPCR with DNA adduct levels

**Gene**	**Cell line**	**Microarray and RTq-PCR**	**Microarray and DNA adducts**	**RTq-PCR and DNA adducts**
		
		**Pearson Correlation Coefficient**		
*AKR1C3*	MCF-7	**0.723**	0.298	0.452
	HepG2	**0.765**	0.138	-0.481
*BAX*	MCF-7	**0.828**	**0.783**	**0.976**
	HepG2	**0.704**	0.302	0.144
*CDKN1A*	MCF-7	**0.947**	**0.953**	**0.968**
	HepG2	**0.634**	**0.694**	**0.791**
*HISTH13D*	MCF-7	**0.967**	**-0.905**	**-0.983**
	HepG2	**0.762**	-0.240	0.082
*IER3*	MCF-7	-0.225	0.243	**-0.921**
	HepG2	**0.879**	**0.764**	0.633
*PCNA*	MCF-7	**0.882**	-0.520	-0.272
	HepG2	**0.784**	0.092	-0.048
*NQO1*	MCF-7	**0.879**	**0.923**	**0.980**
	HepG2	0.419	-0.433	0.617
*CYP1B1*	MCF-7	0.125	-0.045	-0.204
	HepG2	-0.308	-0.537	0.251

^1^Values in bold are significant Pearson correlation coefficients (p < 0.05).

**Table 7:** Mean percentages of the MCF-7 and HepG2 cell cycle parameters after 2.5 μM BaP treatment versus control

MCF-7						
	% G1		% S		% G2	
	
Time (h)	DMSO	BaP	DMSO	BaP	DMSO	BaP

24	66.2	63.8	**24.8**	**29.1**	9	7.2
48	74.3	75.1	12.6	18.8	**13.3**	**6.2**
72	**75.5**	**81.1**	14.6	13.7	10	5.2

HepG2						

	% G1		% S		% G2	
	
Time (h)	DMSO	BaP	DMSO	BaP	DMSO	BaP

24	**73.7**	**63.2**	**15.8**	**27**	10.6	9.9
48	**74.4**	**70.4**	**13**	**19.4**	12.7	10.3
72	76.2	71.5	14.3	13.4	9.6	15.2

The values are representative of two independent experiments for which the standard deviation was never greater than 5%. Bold values indicate a significant difference (p < 0.05) between the BaP- and DMSO-treated cells.
